# Cutaneous Hematologic Neoplasms in Children: Overview and Update

**DOI:** 10.3390/dermatopathology13020024

**Published:** 2026-05-29

**Authors:** Philippe Drabent, Anne Welfringer, Alejandro A. Gru, Thierry J. Molina, Sylvie Fraitag

**Affiliations:** 1 Pathology Department, Hôpital Necker-Enfants Malades, APHP, 75015 Paris, France; 2 Department of Dermatology, Hôpital Necker-Enfants Malades, APHP, 75015 Paris, France; 3 Department of Dermatology and Pathology, Columbia University Irving Medical Center, New York, NY 10032, USA

**Keywords:** skin, children, lymphoma, leukemia, hematologic neoplasms

## Abstract

Cutaneous hematologic neoplasms in children are relatively rare and encompass a wide range of lymphoproliferative and myeloproliferative disorders. This review explores and updates the classification, clinical presentation, diagnostic challenges, histopathology, and management of pediatric lymphomas, lymphoproliferations, and leukemias that may be seen in the skin. The most frequent of them are lymphomatoid papulosis (LyP) and mycosis fungoides (MF), and are discussed first, with a particular focus on differential diagnosis and overlaps with benign lesions—mainly pityriasis lichenoides—which raises questions regarding the delineation of these entities and their potential interconnection. It is important to underline that most cutaneous lymphoproliferations are indolent in children: primary cutaneous CD4+ small/medium T-cell lymphoproliferative disorder, subcutaneous panniculitis-like T-cell lymphoproliferation (non-associated with *HAVCR2* mutations), primary cutaneous marginal zone lymphoproliferative disorder, and EBV-related lymphoproliferative disorders. However, aggressive hematologic malignancies, although rarer, must not be missed; these are mostly leukemias (but not all forms) and blastic plasmacytoid dendritic cell neoplasm. We emphasize the importance of clinical–pathological correlation, with clonality studies playing a crucial role in some cases. Management strategies are briefly reviewed, ranging from skin-directed therapies like phototherapy and corticosteroids to systemic treatments for more aggressive forms of leukemia cutis and lymphomas.

## Simple Summary

Pediatric cutaneous hematologic neoplasms are rare and heterogeneous. The most frequent entities include primary cutaneous CD30-positive T-cell lymphoproliferative disorders, mycosis fungoides, and leukemias. In children, several entities display indolent clinical behavior, supporting the use of the term “lymphoproliferative disorders” rather than lymphoma in selected cases. Diagnosis remains challenging due to overlapping clinical and histopathological features with benign dermatoses, frequent atypical presentations, and limited specificity of clonality studies. Accurate classification relies on careful clinicopathological correlation, integrating age, lesion distribution, histology, immunophenotype, and molecular findings. This review highlights key diagnostic clues and pitfalls across major entities. Improved recognition of these patterns is essential for appropriate management and avoidance of overtreatment and may refine disease classification and prognostic stratification in pediatric dermatopathology.

## 1. Overview of the Last WHO and ICC Classifications: Pediatric Entities in the Skin

Six main series of pediatric skin lymphomas have been published in the last twenty years, the last of which did a great job of reviewing previous data [1]. Taken all together, these series collected 329 cases of pediatric hematologic neoplasms in children. The most frequent type of skin lymphoma is lymphomatoid papulosis (LyP, n = 113), followed by mycosis fungoides (MF, n = 87), cutaneous anaplastic large-cell lymphoma (ALCL, n = 33), cutaneous lymphoblastic lymphomas (LBL, n = 23), unclassified lymphomas (n = 21), primary cutaneous marginal zone lymphoma (or primary cutaneous marginal zone lymphoproliferative disorder, pcMZLPD, n = 18), primary cutaneous CD4+ small/medium T-cell lymphoproliferative disorder (pcSMCLPD, n = 12), cutaneous EBV-related lymphoproliferative disorders (EBV-LPD, n = 6), subcutaneous panniculitis-like T-cell lymhoma (SCPLTL, n = 5), cutaneous extra-nodal NK/T-cell lymphoma, nasal type (EN-NK/TLNT, n = 5), and cutaneous follicle center lymphoma (FCL, n = 3) [1,2,3,4,5,6]. Isolated cases of Sezary syndromes, primary cutaneous γ/δ T-cell lymphomas, and primary cutaneous peripheral T-cell lymphomas NOS have also been reported in two of these series [1,3]. There is an ongoing debate on the diagnostic terminology utilized for primary cutaneous marginal zone lymphoma and subcutaneous panniculitis-like T-cell lymphoma, especially in children, whereas they should be considered lymphomas or lymphoproliferative disorders, and we favor the second denomination in children and in the skin, given the clinical outcome. These semantic distinctions are summarized in Table 1.

Other malignant cutaneous hematologic disorders, such as acute leukemias and blastic plasmacytoid dendritic cell neoplasm (BPDCN), which may be seen in the skin in children, are also included in this review. Cutaneous dissemination in leukemias are mostly acute lymphoblastic leukemias (ALL); however, in neonates and infants, acute myeloblastic leukemias (AML) are more frequent (up to 75% of cases) and may present as a “blueberry muffin baby” [7]. Among ALL, cutaneous specific lesions of B-ALL are approximately 5 times more frequent than T-ALL (again: often revealing the disorder) [8]. In the skin, B-ALL is also more frequent than T-ALL, and about one third of cases present as lymphoblastic lymphomas, with no extra-cutaneous involvement [9]. Blastic plasmacytoid dendritic cell neoplasm (BPDCN) is primarily a disease of elderly patients; however, some cases can occur in children, even in patients less than 1 year of age, and may be part of the differential diagnosis of leukemias [10].

**Clinical–pathological correlation is necessary for the diagnosis of hematologic neoplasms in children, particularly in cutaneous lymphomas.** The histopathologic results should always be evaluated in the context of the patient’s age, topography, clinical examination, blood work-up, clonality, and, in some instances, even molecular alterations. These results are crucial not only for the diagnosis but also for their management. In the present review, we discuss mainly the diagnostic clues, and therefore begin with some important clinical points.

There are interesting differences in age of onset and sex between these different entities. LyP may be seen from as young as 3 months, with a median age of about 7 years at diagnosis [1]. In contrast, MF is seen in older children with a median age of 9 to 12.2 years at diagnosis and 8.6 years at the onset of lesion, and as young as 2 years [1,11,12,13]. This explains why MF cases are over-represented in series including adolescents and young adults [2,5] compared with young children [3]. ALCL has a median age of onset between 8 and 9 years [1]. Lymphoblastic lymphomas are mainly of the B phenotype and are seen in infants and young children, with a median age of about 2 years. Primary cutaneous CD4+ small/medium T-cell lymphoproliferative disorder may be seen as young as 5 years, with a median age between 10 and 11 years [1]. All other lymphomas are seen after the age of 10 years. Interestingly, primary cutaneous follicle center lymphoma is extremely uncommon in children and adolescents [14], where cutaneous marginal zone B-cell lymphoproliferative disorder represents most cases of B-cell lymphomas. As a result of these data, a diagnosis of lymphoma in a neonate or infant is exceptional; most hematologic neoplasms at this early age are lymphoblastic leukemias/lymphomas. Conversely, a diagnosis of MF (all the more of B-cell lymphoma) would be exceptional before the age of 10 years and should only be made with comprehensive evidence.

The overall female-to-male ratio for lymphoproliferative disorders is 1:1.6, due to a striking male predominance in most categories of lymphomas (especially the most frequent ones), except for pcMZLPD, in which girls are over-represented (10 girls for 8 boys). The sex ratio seems balanced in primary cutaneous CD4+ small/medium T-cell lymphoproliferative disorder, EBV-related lymphoproliferative disorders, subcutaneous panniculitis-like T-cell lymphoma, and cutaneous extra-nodal NK/T-cell lymphoma of nasal type, but the number of cases is very low, and it is probably wise not to over-interpret the data [1]. Sezary syndrome has only been reported once in a 17-year-old man and should be considered a T-cell leukemia of adults.

In this article, we will focus on the main pediatric hematologic disorders and their associated findings in children.

## 2. Primary Cutaneous CD30-Positive T-Cell Lymphoproliferative Disorders

The incidence rate of pediatric cutaneous T-cell lymphomas (CTCL), including primary cutaneous CD30^+^ T-cell lymphoproliferative disorders, is estimated between 0.1 and 0.3 per 1 million person-years [15]. The most common subtype of cutaneous hematologic disease in children is lymphomatoid papulosis (LyP). There is male predominance (67% of cases). Very early cases, as young as 3 months, have been described [1,15].

**Figure 1:**
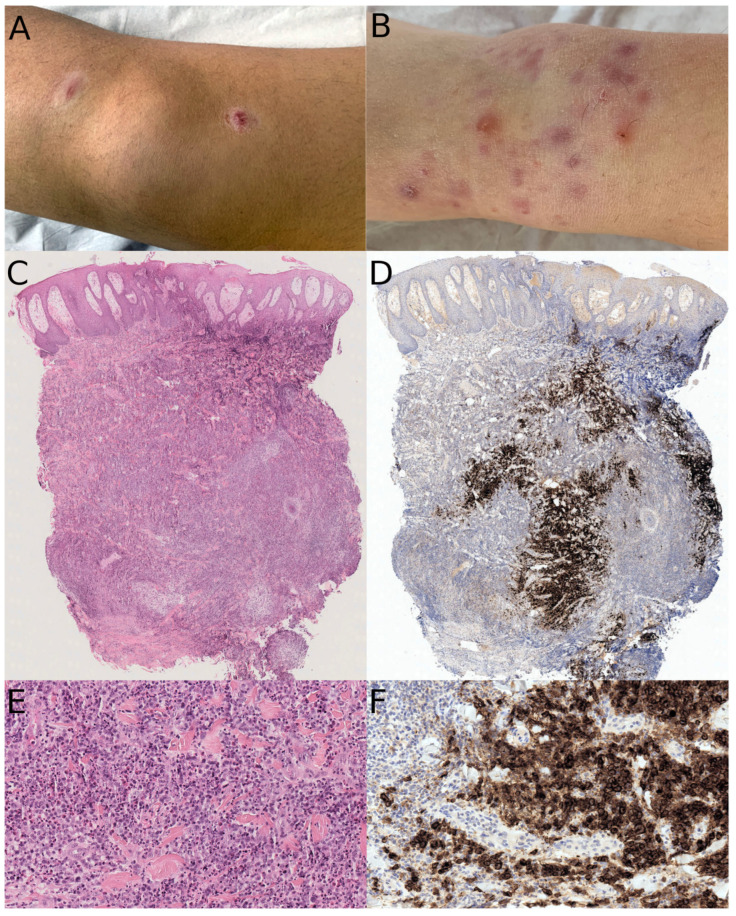
Lymphomatoid papulosis. (**A**,**B**). Clinical picture in two different patients: erythematous and crusted papular lesions on the elbow and knee. (**C**). Whole-slide view showing epidermal hyperplasia, papillary edema, and a deep cellular infiltrate throughout the dermis (H&E). (**D**). Whole-slide view of CD30 immunohistochemistry showing numerous CD30-positive cells. (**E**). Admixture of large lymphoid cells and accompanying inflammatory cells: small lymphocytes and eosinophils (H&E ×200). (**F**). The large lymphoid cells are positive for CD30 (×200). This figure is from the dermatology department at Necker–Enfants Malades Hospital and illustrates the corresponding pathology.

**Figure 2:**
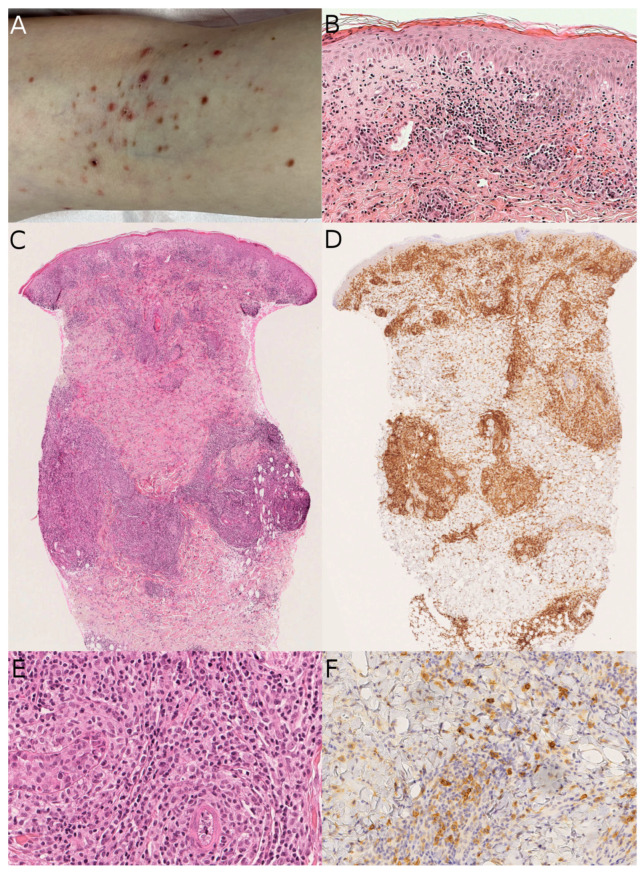
PLEVA-like lymphomatoid papulosis. (**A**). Clinical lesions are macules and papules, some of them crusted, of smaller size and more numerous compared with Figure 1. (**B**). Parakeratosis, interface dermatitis with keratinocyte apoptosis, dermal lymphocytic infiltrate with red blood cells, all reminiscent of Pityriasis lichenoides (H&E ×200). (**C**). At lower magnification, the dermal infiltrate is very deep with nodular architecture at the dermal–subcutis junction, in favor of lymphomatoid papulosis (H&E whole-slide view). (**D**). The lymphoid infiltrate is diffusely positive for CD3. (**E**). High-power examination reveals small lymphocytes but also medium-sized lymphoid cells and some large cells (H&E ×400). (**F**). The larger lymphoid cells are positive for CD30 (×400). This figure is from the dermatology department at Necker–Enfants Malades Hospital and illustrates the corresponding pathology.

LyP is a chronic, waxing and waning, self-healing eruption, with a self-limiting course and a generally benign outcome. Clinically, the lesions present as erythematous papules or papulonecrotic lesions, sometimes ulcerated. The lesions may harbor a brown hue or purpuric features (Figure 1A,B). Sometimes the lesions can be larger and solitary, mimicking cutaneous anaplastic large cell lymphoma (pcALCL). A continuum of lesions that clinically and pathologically overlap between LyP and pcALCL has been reported in some patients (see below, last paragraph of the section, for differential diagnosis with pcALCL). They mainly affect the trunk and extremities, but can occur anywhere. Mucosal lesions are rare. Most patients have no evidence of extracutaneous involvement [1,15]. However, adult patients can have locoregional lymph node involvement [16]. Pruritus may be present [17]. The main differential diagnoses in children are pityriasis lichenoides et varioliformis acuta (PLEVA) or insect bites (Figure 2A). In PLEVA, lesions are more numerous and are generally smaller in size. Treatment is based on therapeutic abstention, dermosteroids, phototherapy, or methotrexate in profuse and necrotic forms. The disease prognosis is good, with excellent survival rates, but a possible association with non-Hodgkin’s lymphoma has been reported, warranting clinical follow-up (namely mycosis fungoides, primary cutaneous or systemic acute myeloid leukemia) [15,18].

The histopathology of LyP may be similar to that of insect bites, scabies, in particular post-scabies nodules, or rarely eosinophilic or neutrophilic dermatoses, when the number of accompanying neutrophils or eosinophils is high. However, PLEVA is usually not a microscopic differential diagnosis, as there are never eosinophils, and the pattern of infiltration in the dermis is different [19]. The inflammatory stroma around molluscum contagiosum can also be mistaken as LyP. The histological appearance of LyP varies widely depending on the subtype. Indeed, six subtypes are usually recognized: type A to type E, and a separate one with a *DUSP22/IRF4* translocation [20]. In children, type A is by far the most frequent (about 52% of cases in the literature, but even more in our practice, probably up to 80%) [1]. None of the subtypes of LyP carry any prognostic significance.

Type A LyP consists of a wedge-shaped dermal infiltrate of large cells with anaplastic features, pleomorphic vesicular nuclei and prominent nucleoli, sometimes multinucleated (“Reed–Sternberg-like”), scattered or in small clusters within a reactive infiltrate of neutrophils, eosinophils, plasma cells, histiocytes and lymphocytes (Figure 1C–F). Epidermotropism of large cells may be seen. Type B is characterized by a prominent epidermotropism of small to medium-sized cells simulating plaque stage mycosis fungoides; type C displays a nodular pattern with numerous large cells similar to primary cutaneous anaplastic large cell lymphoma. The coexistence of different patterns in the same patient that range from type A, B, and C has been reported.

Type D is the second most frequent subtype of LyP in children (25% of cases), characterized by prominent epidermal hyperplasia and epidermotropism with large cells in the epidermis (contrary to type B) and often a CD8-positive phenotype [1]. The particular *DUSP22/IRF4* variant, genetically defined, has not been described in children to date.

In insect bites, the number of eosinophils in the dermis and/or subcutis is usually very high [17]. The degree of CD30 expression is lower compared with LyP and usually lacks the clustering of cells. In challenging cases, T-cell clonality studies can be looked for; close clinical follow-up is also key.

PLEVA can sometimes be difficult to differentiate from LyP, especially when numerous CD30+ T-cells are present. Kempf et al. studied such cases of PLEVA with prominent CD30+ cells mimicking LyP in a series of 13 cases [21]. Those PLEVA cases were characterized clinically by small erythematous macules that evolved into necrotic papules. There was no waxing and waning course. Interestingly, a T-cell clone was found in about 50% of PLEVA cases, which obviates the use of clonality as a criterion for differential diagnosis. The difficulty resides in the occurrence of LyP in patients with an authentic PLEVA history [17]. This raises the question of the link between the two entities and whether they might be the two ends of the same spectrum, ranging from small benign lesions to more nodular and aggressive cases, with possible evolution from one to the other. Indeed, a large study by Cerroni et al., which included pediatric cases, included the term atypical pityriasis lichenoides to recognize this group of challenging cases with overlap between LyP [22] (Figure 2).

Primary cutaneous anaplastic large cell lymphoma (pcALCL) is at the other end of the spectrum of primary cutaneous CD30-positive T-cell lymphoproliferative disorders, with a worse prognosis than LyP, but still a better prognosis than in adults, especially when lesions are solitary or localized. Some of the lesions may regress spontaneously. Multifocal lesions are more worrisome and may be fatal [1]. Most lesions are nodules or papules, often ulcerated. Histologically, the dermis is infiltrated by large tumor cells with anaplastic nuclei, prominent nucleoli, and large cytoplasms. The cells may show an immunoblastic morphology. Especially in ulcerated cases, due to reactive inflammation, the lesion may look like LyP, but there is usually a higher density of CD30-positive cells. However, the most important clue to differentiate pcALCL from LyP is the clinical presentation: solitary or localized nodule (more rarely multifocal) in pcALCL versus multiple waxing and waning papules in LyP. The limit of 2 cm in lesion size, which has been suggested in the last WHO classification of skin tumors, has not been studied specifically in children, to our knowledge.

## 3. Pediatric Mycosis Fungoides

**Figure 3:**
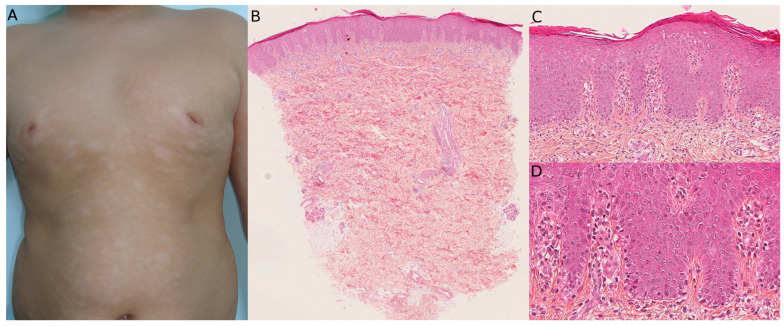
Mycosis fungoides (MF). (**A**). Hypopigmented patches on the trunk of a young boy, typical of hypopigmented MF. (**B**). Whole-slide view showing a psoriasiform epidermal hyperplasia and a band-like superficial lymphocytic infiltrate (H&E). (**C**). Epidermotropism is more visible at higher magnification (H&E ×200). (**D**). However, there is no conspicuous nuclear atypia in the intra-epidermal lymphocytes (H&E ×400). This figure is from the dermatology department at Necker–Enfants Malades Hospital and illustrates the corresponding pathology.

A large case series has estimated that pediatric MF constitutes approximately 4–5% of all age MF cases in the United States. Pediatric MF lesions begin between the ages of 6 and 9 years, with most diagnoses between the ages of 9 and 12 years [12]. The delay in diagnosis of pediatric MF may be due to MF lesions frequently mimicking various benign dermatoses in childhood, such as pityriasis alba, eczema, psoriasis, post-inflammatory hypopigmentation, tinea versicolor, or vitiligo, since hypopigmented presentations are frequent (Figure 3A).

As children mainly show early forms of MF, the patch stage is primarily observed. The most frequent clinical forms of pediatric MF are hypopigmented and folliculotropic (with follicular papules resembling keratosis pilaris, alopecia, and cysts and comedones), then poikilodermatous MF [23]. Hypopigmented MF is more frequent in patients with a high phototype. Most childhood MF are early stages (stage 1a–2a in 91.6–97%) with an indolent course [13,24], and present as erythematous patches and plaques [25,26].

Histologically, MF is an epidermotropic T-cell lymphoma characterized by a proliferation of small to intermediate-sized atypical T-cells. In children, the patch and plaque forms are the most frequent, and this explains why the epidermotropism is usually obvious. In tumor-stage disease, however, epidermotropism may be lost, but these forms are rare in children. Classical features of MF epidermotropism are Pautrier microabscesses (small clusters of neoplastic cells in the epidermis), alignment of tumor cells along the basal membrane, pericellular halos, with no or very slight changes in the epidermis compared with the intensity of the epidermotropism (no hyperplasia, no or subtle spongiosis…). There is usually a band-like infiltrate of tumor cells and reactive lymphocytes in the upper dermis. Cellular atypia is variable with irregular and/or enlarged nuclei. In some cases, and probably most early cases in children, the neoplastic cells are very sparse, and the diagnosis may be difficult, overlooked, and may require clonality analysis (which may be polyclonal due to the very low number of neoplastic cells), and sometimes repeated biopsies (Figure 3B–D). Folliculotropism is present in papular lesions and those with a keratosis pilaris-like appearance. There is a predominance of CD8+ T-cell forms in pediatric MF compared with CD4+ classical forms, particularly in cases with a hypopigmented appearance.

**Figure 4:**
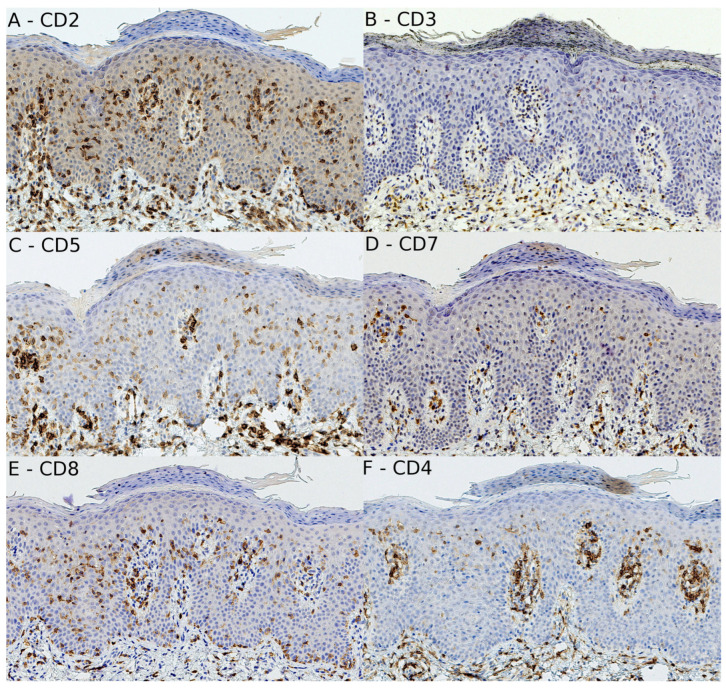
Immunohistochemistry in a hypopigmented CD8-positive mycosis fungoides. (**A**). CD2 is retained (×200). (**B**). Loss of CD3 in the epidermal component (×200). (**C**). CD5 may be partially lost in the epidermal component with a weaker intensity of staining compared with dermal lymphocytes (×200). (**D**). Loss of CD7 in the epidermal component (×200). (**E**). Epidermal lymphocytes are strongly positive for CD8 (×200). (**F**). There is no (or rare) staining for CD4 in epidermal lymphocytes; positive cells in the dermis are histiocytes (×200). This figure is from the dermatology department at Necker–Enfants Malades Hospital and illustrates the corresponding pathology.

Like the varied clinical presentations of MF, there is an endless list of histological differential diagnoses, exemplified by the many histological variants. The hypopigmented variant is the most common in children, as already mentioned (more than 50% of cases), and may be wrongly diagnosed as vitiligo or pityriasis lichenoides chronica (see next section). This variant is almost always made of CD8+ T-cells (Figure 3 and Figure 4). The folliculotropic variant (up to 30% of cases in some series) and poikilodermatous variant are less challenging. It has been shown that cases of benign follicular mucinosis do not progress to MF in children, and we believe that the folliculotropic variant of MF with mucinosis does not exist in children (we have never come across such a case) [27]. Some uncommon variants may challenge the pathologist: palmoplantar MF, pigmented purpuric dermatosis-like variant, granulomatous variant, ichthyosiform variant, inflammatory linear verrucous epidermal nevus-like variant, and pagetoid reticulosis, to name a few [11,12]. In those cases, the loss of one or more of the pan-T antigens (mostly CD7) and the presence of a T-cell clone are invaluable for achieving the right diagnosis. More recently, the use of TRBC1 immunohistochemistry in the routine diagnosis of T-cell lymphomas has facilitated the recognition of clonal T-cells by immunohistochemistry [28,29]. Importantly, one must always bear in mind that some benign dermatoses may be associated with T-cell monoclonality, so that the assessment of T-cell receptor rearrangement is not, on its own, sufficient for a diagnosis of MF. It is the case in PL, as already mentioned, but also in benign follicular mucinosis or lichenoid purpura.

Although rare, cases of transformation, defined as the presence of more than 25% of large cells with nuclei more than four times the normal size, among the neoplastic cells, and death have been reported in children (3.9%), which justifies the long-term follow-up, and particularly histopathologic reassessment when progression of clinical lesions develops. Factors associated with a poorer prognosis include a longer time from onset to diagnosis, granulomatous slack skin, granulomatous MF, a history of organ transplant, and stage 2 disease at the time of diagnosis [13]. A recent consensus has added specific severity criteria for children, in addition to the tumor-node-metastasis-blood staging: pruritus, functional or esthetic impairment (e.g., palms, soles, genitalia), quality of life impact, and psychological aspects (e.g., embarrassment, anxiety, depression), including parental anxiety [24]. The hypopigmented and poikilodermatous variants appear to have a better prognosis. CD8+ T-cell immunophenotype is also associated with a better prognosis compared with adult-onset MF.

Differential diagnosis between MF and pityriasis lichenoides chronica (PLC) might be challenging [23]. Recent evidence suggests a relationship between pityriasis lichenoides (PL) and MF, categorized into three groups: PL with T-cell infiltration, PL evolving to MF, and PL-like MF. PL-like MF is a recently described subtype of MF. These lesions can lead to widespread hypopigmentation. The clinical presentation is similar to that of PLC, while its histopathological and immunophenotypic findings are supportive of MF [30].

As already mentioned in the section about LyP, a T-cell clone may be found in PL, including PLC and PLEVA, in up to 50% of cases. Therefore, clonality is not useful in the differential diagnosis between MF and PLC. Positive clonality studies can also be identified by NGS [31]. Matching clones were identified in 2 patients with an overall classic presentation of PLC, in the absence of progression to MF. In cases with a loss of one pan-T antigen, a diagnosis of MF can be favored. However, in some cases, it may be impossible for the pathologist to settle on one diagnosis or the other. If the diagnosis is not certain, follow-up with iterative biopsies is recommended.

First-line treatment in children, because of the early and indolent presentation, is based mostly on skin-directed therapies, i.e., light, topical corticosteroids (TCS), but in corticoresistant cases, other therapies can be used: bexarotene, nitrogen mustard, vitamin D analogs, carmustine, pimecrolimus, or phototherapy [12].

## 4. Primary Cutaneous CD4+ Small/Medium T-Cell Lymphoproliferative Disorder

Clinically, pcSMCLPD usually presents as a solitary papule or nodule on the face, neck, and upper limbs [32,33]. In children, atypical forms are described: multiple red-brown to yellow purpuric MF-like plaques that lack desquamation, or multiple papules [1]. The lesions may appear early, starting from 3 months of age. They follow an indolent clinical course, which explains the denomination of “lymphoproliferative disorder” [34].

Histologically, pcSMCLPD is a dermal proliferation of uniform small to medium-sized T lymphocytes with pale, scanty cytoplasm and hyperchromatic nuclei, which are arranged in a bandlike, nodular, or diffuse architecture, with no or only focal epidermotropism. In children, plaque-like forms with only superficial infiltration of the dermis may be seen, while deeper infiltration, which can involve the subcutis, is less frequent. Accompanying lymphocytes, plasma cells, neutrophils, or eosinophils may be present. The tumor cells have a T-follicular helper (TFH) phenotype, expressing PD1 and BCL6, sometimes CXCL13 or ICOS, but usually not CD10. They are obviously CD4-positive and may have loss of CD7 or, more rarely, CD2 or CD5. There is usually a rich CD20-positive B-cell population that may also be CD30-positive and is surrounded by the PD1-positive T lymphocytes. Some cases of pcSMCLPD can have overlap features with pcMZLPD [35]. There is T-cell clonality, and a coexisting B-cell clone may occur in 26% of cases.

It is important to be aware of this diagnosis, which is a benign lymphoproliferation, to avoid wrongly diagnosing a variant of CD4+ MF (and especially a tumor stage MF in deep lesions). In this spirit, a full body skin exam is, of course, mandatory to make sure no MF patches are present, which would argue against the diagnosis of pcSMCLPD. Again, the clinical history is different between the two diseases, and any atypia for MF should prompt the realization of TFH antibodies. The minimal TFH markers are PD1 and BCL6. It is better to have a third marker if possible. In our experience, CXCL13 is easier to tune and more robust than ICOS.

## 5. Subcutaneous Panniculitis-like T-Cell Lymphoma

**Figure 5:**
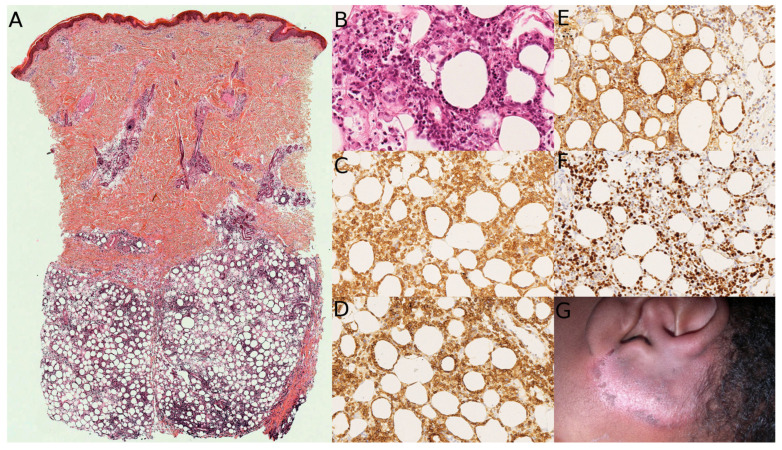
Subcutaneous panniculitis-like T-cell lymphoproliferation. (**A**). Low-power view showing intense lobular panniculitis (H&E whole-slide view). (**B**). At high magnification, there are atypical lymphoid cells with abundant apoptotic debris and rimming of adipocytes (H&E ×400). (**C**). Diffuse positivity for CD3 (×200). (**D**). Diffuse positivity for CD8 (×200). (**E**). Diffuse positivity for granzyme B (×200). (**F**). High proliferation index (Ki67 ×200). (**G**). Clinical picture: deep indurated plaque in the pre-auricular area. This figure is from the dermatology department at Necker–Enfants Malades Hospital and illustrates the corresponding pathology.

Subcutaneous panniculitis-like T-cell lymphoma (SCPLTL) is an uncommon form of cutaneous lymphoma in the pediatric population [36]. The median age of onset is 11.1 years [37]. Clinically, the lesions are painless subcutaneous nodules or plaques (Figure 5G), most commonly involving the lower extremities and/or trunk. They are often triggered by infection or autoimmune disorders (e.g., lupus erythematosus, juvenile rheumatoid arthritis, etc). There are probably two different forms of the disease: benign forms, which should be named “lymphoproliferation” rather than “lymphoma”, and which may regress spontaneously, and forms that are associated with hemophagocytic syndrome. In this latter form, the condition may be complicated by systemic involvement [38], in particular in patients harboring germline mutations in the *HAVCR2* gene, altering T-cell immunoglobulin mucin 3 (TIM3) protein [39]. These children have a higher risk of hemophagocytic syndrome [40,41]. The difference between the two forms is clinical and, even in the absence of clinical data, we would suggest a “lymphoproliferation” terminology in children, coupled with a study of the *HAVCR2* gene or TIM3 immunohistochemistry, if available.

SCPLTL can be misdiagnosed as lupus panniculitis. Indeed, 19% of patients with SCPLTL have an associated autoimmune disease, including systemic lupus erythematosus in most cases [42]. SCPLTL presents as a mainly lobular atypical infiltrate of small to medium-sized lymphocytes, with only scattered large cells. Rimming of individual adipocytes is typical but may be focal and is not specific (Figure 5A,B). There is almost always associated histiocytes, with fat necrosis and karyorrhexis (apoptotic debris). Reactive small lymphocytes may also be seen, but neutrophils or eosinophils are rare. Contrary to lupus panniculitis, plasma cells and lymphoid follicles are sparse. However, there is a real overlap between the two entities in some cases.

The neoplastic cells of SCPLTL have a cytotoxic phenotype with expression of CD8 and TCRab. They are most often CD3+, CD8+, CD4-, Granzyme B+, TIA-1+ and perforin-positive (Figure 5C–F). There is no need to perform all cytotoxic markers; one should be enough. The pan-T antigens that are most often lost are CD5 and CD7. Ki67 always shows an increased proliferation index, and TCRgd must be negative. EBV is also negative. Clusters of CD123+ plasmacytoid dendritic cells are more frequently seen in lupus panniculitis but can also be seen in STPCL. In benign forms, the treatment is based on immunosuppressive agents: corticosteroid alone, or a low dose of methotrexate, cyclosporine A, or hydroxychloroquine [43].

## 6. Lymphoblastic Lymphomas/Leukemias

Lymphoblastic lymphomas/leukemias (LBL) are divided into two categories: T-lymphoblastic lymphoma/leukemia (T-LBL) and B-lymphoblastic lymphoma/leukemia (B-LBL). B-LBL are much more frequent than T-LBL in children [8]. The median age at time of diagnosis of initial skin involvement is 2 years. In leukemic cases (acute lymphoblastic leukemia, ALL), there is often associated hepatosplenomegaly. In lymphomatous cases, characterized by the absence of peripheral blood involvement and less than 25% of blasts in the bone marrow, the most frequent associated clinical sign is the presence of cervical lymph nodes [9].

T-LBL classically presents with a mediastinal mass, lymphadenopathy, and bone marrow involvement. If present, skin lesions are usually multiple nodules, more rarely papules, purpura, ecchymosis, macules, and plaques. These lesions are mostly found on the scalp, face, and trunk.

Patients with cutaneous B-LBL usually present with a large, solitary, firm, painless nodule located mostly, interestingly, on the head and neck region. A bluish color is frequently described [44]. Most children present with a localized disease at first, but bone marrow involvement, lymph nodes, and osteolytic bone lesions are possible. The disease is highly aggressive but may be curable if treated early. Rapid diagnosis is therefore essential [45].

**Figure 6:**
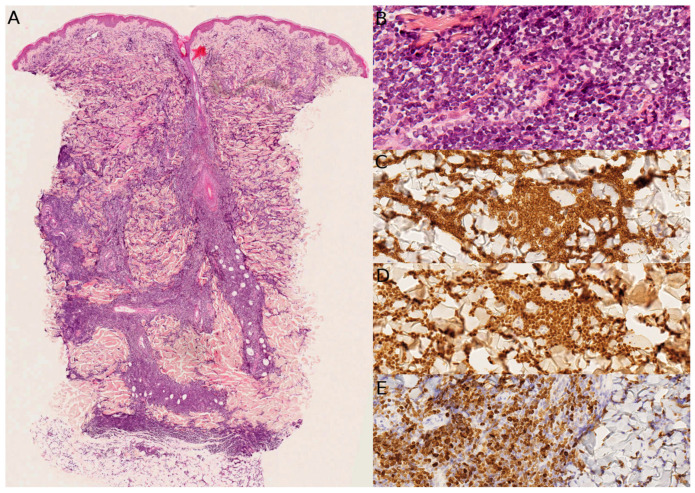
Lymphoblastic lymphoma. (**A**). Extensive superficial and deep lymphoid infiltrate in the dermis, sparing the papillary dermis (grenz zone), with a striking interstitial and peri-adnexal distribution (H&E whole-slide view). (**B**). Monomorphic small lymphoid cells between the collagen bundles (H&E ×400). (**C**). Here, the lymphoblastic lymphoma is of the T phenotype, with diffuse CD3 positivity (×200). (**D**). Diffuse nuclear positivity for TdT (×200). (**E**). High proliferation index (Ki67 ×200). This figure is from the dermatology department at Necker–Enfants Malades Hospital and illustrates the corresponding pathology.

B and T-ALL typically show a monotonous infiltrate of blasts in the dermis and subcutis, dissecting between the collagen bundles, leaving a spared zone (“grenz zone”) in the papillary dermis (Figure 6A). The cells are medium-sized blasts with finely dispersed chromatin and inconspicuous or small nucleoli (Figure 6B). Mitoses are numerous. There is no reactive inflammatory cell. There is consistent expression of TdT (Figure 6C–E). In B-LBL, Pax5 or CD79a are more often expressed than CD20. CD10 is frequently positive. CD99 is almost always positive and may be misleading; it should not be taken as proof for a peripheral neuro-ectodermal tumor.

## 7. Other B-Cell Lymphomas

According to the WHO-European Organization for Research and Treatment of Cancer (WHO-EORTC), primary cutaneous B-cell lymphomas are classified into three major subtypes: primary cutaneous marginal zone lymphoma, primary cutaneous follicle center lymphoma, and primary cutaneous diffuse large B-cell lymphoma, leg-type [46]. This latest form has not been reported in children (See also Table 1).

In children, primary cutaneous B-cell lymphomas are rare (0.93% of cases diagnosed before 20 years of age), and the median age is 16.5 years [47]. The most frequent subtype, almost always seen in adolescents, is primary cutaneous marginal zone lymphoma/lymphoproliferative disorder (pcMZLPD) in 77% of cases. The most common primary sites are the face, scalp, and neck, and the upper limbs. This is different from adult cases, so that the topography of lesions (face vs. trunk) does not help differentiate between pcMZLPD and primary cutaneous follicle center lymphoma. Lesions are red/violaceous papules, plaques, or nodules. The course of pediatric pcMZLPD is indolent with excellent prognosis. The current ICC denomination for this entity favors the term ‘primary cutaneous marginal zone lymphoproliferative disorder’.

**Figure 7:**
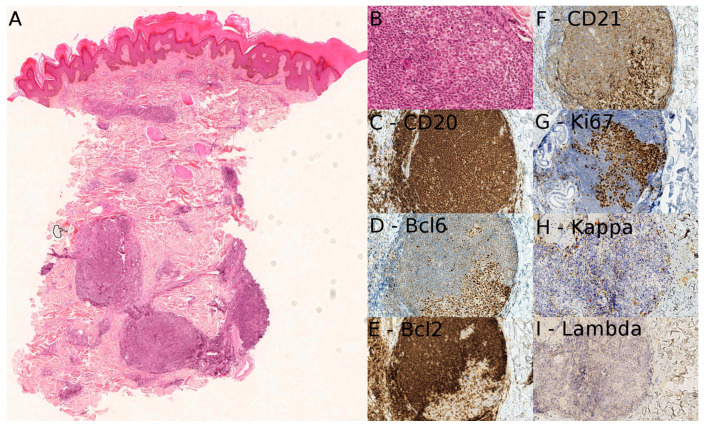
Primary cutaneous marginal zone lymphoproliferative disorder. (**A**). Low-power view showing the nodular architecture of the lymphoproliferation (H&E whole-slide view). (**B**). At high magnification, there is follicular disarray with infiltration by small-sized monomorphic lymphocytes (H&E ×200). (**C**). Diffuse CD20 positivity (×200). (**D**). The small cells are negative for Bcl6, with positive control on the remaining germinal center cells (×200). (**E**). The small cells are positive for Bcl2 (×200). (**F**). There is disruption of the follicular dendritic cells network (CD21 ×200). (**G**). The small cells show a low proliferation index compared with the remaining follicle center cells (Ki67 ×200). (**H**,**I**). There is a kappa light chain monotypia, with no positivity for lambda (×200). This figure is from the dermatology department at Necker–Enfants Malades Hospital and illustrates the corresponding pathology.

Pathologically, pcMZLPD shows a nodular and/or diffuse pattern of infiltration in the superficial and deep reticular dermis and occasionally extending to the subcutaneous fat (Figure 7A). A periadnexal pattern is typical. A grenz zone is present. There are reactive or atrophic germinal centers and mantle zones surrounded by a paler infiltrate of marginal zone cells. These cells often have a distinctive pale cytoplasm and irregular nuclei (centrocyte-like) with dense chromatin and inconspicuous nucleoli. Follicular colonization by the tumor cells is often evident, but there are few mitoses (Figure 7B). Lymphoplasmacytoid cells and mature plasma cells are present. The tumor cells are CD20+ (and positive for other B-cell markers), BCL2+, CD10-, CD23-, BCL6-. There is usually a monotypic light chain expression, and the cells are class-switched, expressing IgG rather than IgM. CD21 is helpful to highlight the colonization of follicle centers (Figure 7C–I). *IGH* or *IGK* gene rearrangement studies are always favored to be performed in the pediatric age group. However, the identification of clonal plasma cells or B-cells by newer methods (like ultrasensitive bright-field RNA in situ hybridization (BRISH)) can be helpful in the diagnosis [48,49].

A common diagnostic pitfall is the so-called pseudo-lymphoma associated with Lyme disease, which can appear very similar to a primary cutaneous B-cell lymphoma histologically. However, these lesions respond to doxycycline [50].

Primary cutaneous follicle center lymphoma is even rarer in adolescents [14,51], and there is no histopathological difference to its adult counterpart.

## 8. EBV-Related Lymphoproliferative Disorders

EBV-associated T- and NK-cell lymphoproliferative disorders include two systemic diseases:

- Chronic active EBV disease, systemic (CAEBV).
- Systemic EBV-positive T-cell lymphoma of childhood.

They also include two primarily cutaneous forms: hydroa vacciniform lymphoproliferative disorder (HV) and severe mosquito bite allergy [52,53].

**Figure 8:**
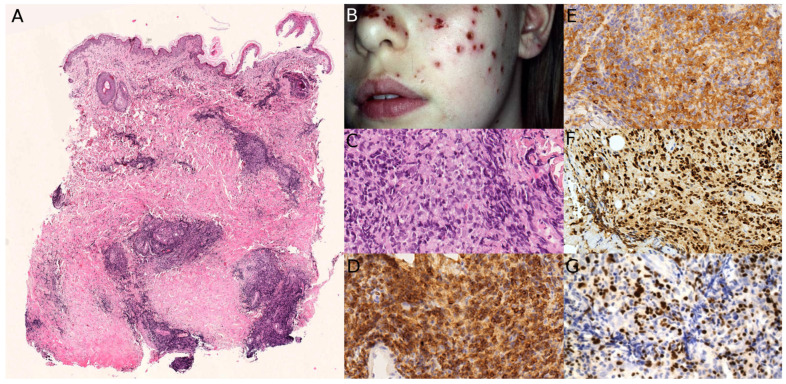
EBV-related lymphoproliferative disorder—hydroa vacciniform type. (**A**). Low-power view showing focal epidermal necrosis and bullae, and a dense perivascular and syringotropic lymphoid infiltrate (H&E whole-slide view). (**B**). Ulcerated lesions on the face of a young boy. (**C**). High magnification reveals a mixture of small and medium-sized lymphocytes with very few large cells in this case (H&E ×400). (**D**). Diffuse CD3 positivity (×400). (**E**). Diffuse CD4 positivity (×400). (**F**). High proliferation index (Ki67 ×400). (**G**). In situ hybridization with an EBER probe: strong positivity (×400). This figure is from the dermatology department at Necker–Enfants Malades Hospital and illustrates the corresponding pathology.

Hydroa vacciniform-like lymphoproliferative disorder (HV) is a primarily cutaneous form of chronic active EBV disease, UV-induced, but the lesions can occur in non-sun-exposed areas. Most cases are described in Asia and central or South America, but there are also cases in Europe and North Africa. The median age at diagnosis is 8 years. Classical HV is characterized by an erythematous eruption in sun-exposed areas associated with papules, vesicles, crusts, and vacciniform scars, with a classical waxing and waning course (Figure 8B). HV presents a risk of progression to systemic lymphoma, and a follow-up is necessary. One case of severe atypical HV-like lymphoproliferative disorder in a 14-year-old girl with hyper IgE syndrome due to *DOCK8* gene mutation has been reported [54].

Severe mosquito bite allergy is a cutaneous manifestation of chronic active EBV infection precipitated by mosquito bites. The median age is 6.7 years old. Clinically, it presents as erythematous papules, plaques, bullae, and ulcers. Systemic symptoms are common (fever, lymphadenopathy, cytolysis). The systemic forms are exceptional.

Pathologically, the lesions of HV consist of a perivascular and periadnexal atypical lymphoid infiltrate, with variable density, and a variable number of large lymphocytes with irregular nuclei. These large cells may be absent in some cases or prominent in others. There may be angiodestruction (Figure 8A,C). There is no epidermotropism, but often vesiculation or ulceration of the epidermis. The abnormal cells are either of T phenotype (60–70%) (they may be CD8+, CD4+, or double positive) or of CD56-positive NK phenotype (30–40%), and are positive for EBV by in situ hybridization (Figure 8D). They represent only a minority of the infiltrate, most lymphocytes being reactive. In some cases, eosinophils are prominent, and those cases are more often of the NK phenotype, with more frequent panniculitis, and also a possible association with hypersensitivity to mosquito bites. In the absence of clinical data, the differential diagnosis with LyP may be an issue. In our experience, the presence of angiocentrism or angiodestruction is an important sign in favor of HV, given the rarity of the “angiocentric” type E LyP in children, and should be a clue to perform EBER in situ hybridization. If clinical data is available, the topography of the lesions in photo-exposed areas is an important clue for HV.

In severe mosquito bite allergy, there is a variably dense infiltrate of small to large atypical lymphocytes with a perivascular distribution in the dermis and subcutis. Angiocentrism and angiodestruction are typically present in more advanced lesions and are responsible for secondary necrosis, ulceration, and bulla formation. Most cases are of the NK phenotype. NK cells are negative for surface CD3 by flow cytometry, and T-cell receptor proteins, and positive for CD3, CD16, CD56, and cytotoxic markers by immunohistochemistry. Lymphoid cells are positive for EBER by in situ hybridization. To our knowledge, there is no histological difference between severe mosquito bite allergy and NK-type HV cases, and the clinical picture is probably the most reliable clue.

## 9. Acute Myeloid Leukemia

**Figure 9:**
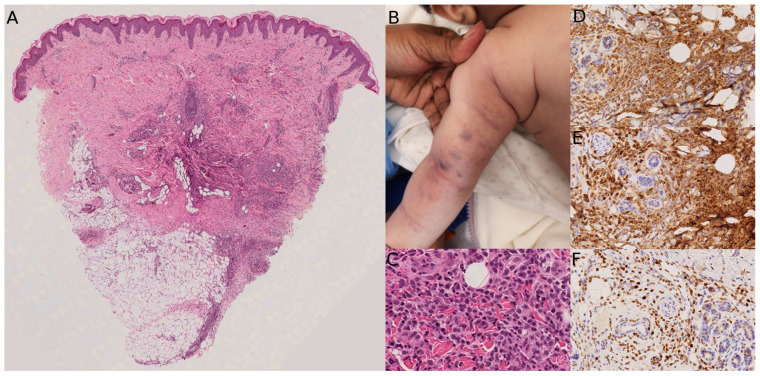
Myeloblastic leukemia. (**A**). The biopsy was taken at the edge of a lesion: the right half of the biopsy is infiltrated by numerous cells in an interstitial pattern, occupying the dermis and subcutis (H&E whole-slide view). (**B**). Bluish or violaceous lesions on the arm of a neonate. (**C**). High-power view shows a rather monomorphous proliferation of medium-sized cells with homogeneous chromatin or small nucleoli, and some visible cytoplasm (H&E ×400). (**D**). Diffuse positivity for CD68 (×200). (**E**). Diffuse positivity for myeloperoxidase (×200). (**F**). High proliferation index (Ki67 ×200). This figure is from the dermatology department at Necker–Enfants Malades Hospital and illustrates the corresponding pathology.

Acute myeloid leukemia (AML), as already mentioned, is more frequent than ALL/LBL in neonates and infants (see part 6 for lymphoblastic lymphoma/leukemia). Clinically, neonatal lesions appear as firm, violaceous nodules, also known as “blueberry muffin baby”. Differential diagnoses in neonates are other causes of “blueberry muffin baby” (Figure 9B) (intrauterine infections of the TORCH group, hemolytic disease of the newborn, Langerhans cell histiocytosis, cutaneous neuroblastoma, and some other small blue round cell tumors) [55,56]. Interestingly, cases of aleukemic leukemia cutis have been reported: these are most often the initial phase of a real leukemia, but some rare cases may spontaneously regress [57]. The total WBC count and bone marrow smear may be normal for a while; therefore, the dermatologist and dermatopathologist may be the first to ascertain the diagnosis. The predominant subtypes of AML in neonates are myelomonocytic, monocytic, and megakaryocytic leukemia (French–American–British [FAB] classification M4, M5, and M7, respectively) [55].

In older children, AML often involves the gums, with gingival hyperplasia as a key symptom. On the skin, it can present as infiltrated plaques or nodules, more rarely papules. There is a male predominance (about 70%), and children are younger than 10 years, and even younger than 2 years in about 2/3 of cases [58,59]. There is CNS involvement in one-third of cases. *MLL*-gene rearrangements are significantly more frequent in cases of AML with skin lesions (46% of *MLL*-gene rearrangements). The most frequently mutated genes are *KRAS*, *KIT*, and *PTPN11*. The most common AML subtypes are FAB M5 (63%), M2 (13%), and M4 (8%) [58].

The histopathologic changes typically include a malignant mononuclear infiltrate of blasts arranged in a diffuse and interstitial pattern between the collagen bundles, leaving a “grenz zone” in the papillary dermis and extending into the subcutis (Figure 9A). The neoplastic cells are medium-sized with a high nuclear-cytoplasmic ratio, numerous mitoses, and caryorrhexis (apoptotic debris) (Figure 9C). As many of the acute myeloid leukemias with skin involvement have a monocytic differentiation, the neoplastic cells are positive for CD68, CD43, lysozyme, and myeloperoxidase (the latter in myelomonoblastic leukemia). The proliferation index is very high with 90 to 100% of positive cells for Ki67 (Figure 9D–F). CD163 is often negative since the neoplastic cells are immature. CD34 is also negative.

Clinically, leukemia cutis in AML is associated with an aggressive clinical course and poor outcome; on rare occasions, the lesions may regress spontaneously. It seems that this behavior depends mostly on genetic anomalies. Cytogenetics are helpful to decipher the degree of severity of the disease, with, for example, 11q23 translocations (responsible for *MLLT4-KMT2A*, *MLLT10-KMT2A*, or *ABI1-KMT2A* fusions transcripts to cite a few) or monosomy 7 in some aggressive forms, and on the contrary, t(8;16) in some spontaneously regressive forms [60,61].

## 10. Blastic Plasmacytoid Dendritic Cell Neoplasm

Blastic plasmacytoid dendritic cell neoplasm (BPDCN) is a rare and aggressive hematologic malignancy that often involves the skin. Since there are mostly case reports and small series in children, it is not easy to determine the mean age at diagnosis. After a review of the literature, it seems to fall between 8 and 10 years. Neonatal and even congenital cases have also been reported. The skin lesions can be a single violaceous nodule, diffuse ecchymotic patches, or multiple subcutaneous nodules [62,63].

BPDCN resembles cutaneous leukemia cutis on histopathologic examination, with a monotonous dermal infiltrate that may extend into the subcutis but spares the epidermis. The infiltrate is made of medium-sized cells that are usually arranged in sheets between the collagen bundles. They have a monocytoid appearance but have the distinctive features of immature blasts (fine chromatin, irregular nuclear borders, prominent nucleoli). The immunophenotype is usually CD4+, CD56+, and most importantly, positive for the plasmacytoid dendritic markers CD123, BDCA2, TCL1, and TCF4. CD123 may be positive in myelomonocytic leukemia; therefore, at least one other antibody (BDCA2, TCL1, or TCF4) must be used as a more specific marker. Some cases may be negative for CD4 and/or CD56. TdT is often positive. CD68 is positive in most cases. BPDCN must be negative for the myeloid markers lysozyme and MPO.

Pediatric BPDCN is clinically less aggressive but often has more dissemination at presentation than adult cases [10]. Given the small number of cases in children, there is no current guideline for treatment. Therapies based on high-risk ALL regimens with multi-drug combinations and maintenance, and central nervous system prophylaxis, have been tried with good outcomes. There is also a place for hematopoietic stem cell transplantation, but the optimal timing in children is unclear [10].

## 11. Conclusions

Pediatric cutaneous hematologic neoplasms require a nuanced diagnostic approach that integrates clinical, histopathological, and molecular data. Table 2 provides an overview of the differential diagnoses according to age. Recognizing their often indolent nature and frequent overlap with benign conditions is critical to avoid misdiagnosis and inappropriate treatment. Refinement of diagnostic criteria and terminology, particularly distinguishing lymphomas from lymphoproliferative disorders, will improve patient management. Future studies should focus on better defining disease biology, prognostic markers, and standardized classification to optimize care in this rare population.

## Figures and Tables

**Table 1:** Comparison of neoplasms’ nomenclature between the International Consensus Classification (ICC) of Myeloid and Lymphoid Neoplasms (2025) and the World Health Organization (WHO) classification of Hematolymphoid Tumors (5th ed.). Main differences are in bold letters.

ICC Nomenclature	Corresponding WHO Entity
Mature T-cell and NK-cell neoplasms	
Epstein–Barr virus-positive T- and NK-cell **lymphoproliferative disorders** of childhood Includes: - Hydroa vacciniform lymphoproliferative disorder - Severe mosquito bite allergy	EBV-positive T-cell and NK-cell **lymphoid proliferations and lymphomas** of childhood Includes: - Hydroa vacciniform lymphoproliferative disorder - Severe mosquito bite allergy
Extranodal NK/T-cell lymphoma, **nasal type**	Extranodal NK/T-cell lymphoma
Mycosis fungoides	Mycosis fungoides
Sezary syndrome	Sezary syndrome
Primary cutaneous CD30-positive T-cell lymphoproliferative disorder - Lymphomatoid papulosis - Primary cutaneous anaplastic large cell lymphoma	Primary cutaneous CD30-positive T-cell lymphoproliferative disorder - Lymphomatoid papulosis - Primary cutaneous anaplastic large cell lymphoma
Primary cutaneous CD4-positive small/medium T-cell lymphoproliferative disorder	Primary cutaneous CD4-positive small or medium T-cell lymphoproliferative disorder
Subcutaneous panniculitis-like T-cell lymphoma	Subcutaneous panniculitis-like T-cell lymphoma
Primary cutaneous gamma-delta T-cell lymphoma	Primary cutaneous gamma-delta T-cell lymphoma
Mature B-cell neoplasms	
Primary cutaneous marginal zone **lymphoproliferative disorder**	Primary cutaneous marginal zone **lymphoma** - Class-switched form - Non-class-switched form
Primary cutaneous follicle center lymphoma	Primary cutaneous follicle center lymphoma
B-lymphoblastic leukemias/lymphomas (B-ALL/BLL)	
T-lymphoblastic leukemias/lymphomas (T-ALL/TLL)	
Pediatric or germline mutation-associated disorders	
Juvenile myelomonocytic leukemia	
Juvenile myelomonocytic leukemia-like neoplasms	
Noonan syndrome-associated myeloproliferative disorder	
Acute Myeloid Leukemias	
Blastic plasmacytoid dendritic cell neoplasm	

**Table 2:** Cutaneous hematologic neoplasms in children according to age. When a disease may occur in several age groups, bold type indicates the age group in which it most commonly presents.

Neoplasms That May Be Congenital or Neonatal	Neoplasms That Are Mainly Seen Before Adolescence	Neoplasms of Adolescents/Young Adults
	**CD30-positive T-cell lymphoproliferative disorders (mostly lymphomatoid papulosis)**	CD30-positive T-cell lymphoproliferative disorders
	Mycosis fungoides	**Mycosis fungoides**
	Primary cutaneous CD4+ small/medium T-cell lymphoproliferative disorder	
Subcutaneous panniculitis-like T-cell lymphoproliferation		
	Lymphoblastic lymphomas	
		Primary cutaneous marginal zone lymphoproliferative disorder
		Primary cutaneous follicle center lymphoma
	Severe mosquito bite allergy	
	Hydroa vacciniform lymphoproliferative disorder	
**Myeloblastic leukemias**	Myeloblastic leukemias	
		Blastic plasmacytoid dendritic cell neoplasm (rare, usually a neoplasm of the elderly)

## Data Availability

No new data was created or analyzed in this study. Data sharing is not applicable to this article.
